# Editorial: Executive Functions in Psychiatric Disorders

**DOI:** 10.3389/fpsyg.2017.01461

**Published:** 2017-09-05

**Authors:** Leandro F. Malloy-Diniz, Débora M. Miranda, Rodrigo Grassi-Oliveira

**Affiliations:** ^1^Molecular Medicine, Pediatrics, Universidade Federal de Minas Gerais Belo Horizonte, Brazil; ^2^Ilumina Neurosciences and Mental Health Belo Horizonte, Brazil; ^3^Developmental Cognitive Neuroscience Research Group (GNCD), Psychology, Pontifícia Universidade Católica do Rio Grande do Sul Porto Alegre, Brazil

**Keywords:** executive functions, psychiatric disorders, neuropsychology, psychometrics, neuropsychological assessments

There's no consensus concerning the definition of Executive functions (EFs), its components and neurobiological underpinnings. Nonetheless, despites of these theoretical disagreements, an essential characteristic of these cognitive processes is its relationship with the capacity to manage cognition, behavior, emotions, and direct the response to established goals.

Diamond (2013) proposes that EFs presents a hierarchical structure with three core processes sub-serving another more complex cognitive functions. According to Diamond, based on previous research of Miyake et al. (2000), the core executive functions are working memory, cognitive flexibility, and inhibitory control. These functions emerge early in development and are the foundation for the elaboration of the complex EFs as problem-solving, planning, reasoning, and abstract thinking. Diamond propositions is a prominent theoretical model but do not focus on issues such as the role of emotion, motivation, and another process like affective decision-making that are frequently considered as a component of executive functions. For example, some authors argued that there are at least two main types of EFs, cool executive functions, related to abstract thinking and hot executive functions more related to emotion and motivation (Zelazo and Carlson, 2012).

According to Johnson (2012), EFs are expected to be impaired in psychiatric disorders. Therefore, these patients usually present a great chance of prejudice in adapt to the demands of social, workplace, school, and other contexts. Deficits in executive functions are related to marital stress (Bouchard and Saint-Aubin, 2014), suicide (Malloy-Diniz et al., 2009), lack of adherence to treatment (Perez et al., 2016), and poor academic performance (Ribner et al., 2017). Therefore, the understanding of the relationship of executive functions and psychopathology is necessary to improve clinical management of psychiatric patients. The primary objective of this research topic, which includes 13 articles from 70 authors from several fields of knowledge, was just recently described in a series of studies about executive functions in psychiatric.

Considering the theoretical perspective, Kluwe-Schiavon et al. provide a comprehensive discussion revisiting traditional EFs definitions. Authors propose that it is necessary to transcend the hot and cool dichotomy, and consider EFs in a dynamic and dimensional perspective.

Most of the articles included in this research topic discussed the relationship between EFs and psychiatric conditions. Duijkers et al. presents a review examining the relationship between executive functions deficits and self-regulation in those patients who suffered by dual pathology (substance use disorder and at least a comorbid disorder). The authors stressed the need to include the executive function assessment and treatment in the clinical management of these patients. Still considering the relationship between substance use disorders and EFs, Witter et al. reports results reinforcing the knowledge about the relationship between impulsivity/risk taking behavior and cocaine use. Authors were also presenting an important result that risk decisions in cocaine users could be related to an underlying mechanism that impedes the learning from immediate mistakes.

Soraggi-Frez et al. presents evidence that working memory deficits is often presented in Bipolar Disorder even in the euthymic stage. Furthermore, authors argue that the hedonic detector, a new component of Baddley's Working Memory model is frequently deregulated interfering in the affective judgment of environmental stimuli and experiences. Caixeta et al. also discussed cognitive Deficits in Bipolar patients. In a study of cognitive deficits in elderly depressed bipolar patients, authors found that neuropsychological deficits are comprehensive including working memory, processing speed, inhibitory control, and cognitive flexibility impairment. Together, Soraggi-Frez et al. and Caixeta et al. add evidence to the scientific literature concerning executive functioning deficits in Bipolar Disorder independently of age and current affective status. Finally, still in the field of executive functions in mood disorders, Almondes et al. present an interesting report that in elderly the interaction between depression and sleep complains contributes to a worse performance in EFs Tasks.

Executive function deficits were also discussed in two other psychopathologies, schizophrenia and ADHD. Berger et al. studied the electrophysiological pattern of activation comparing healthy control and two groups of schizophrenic patients (clustered according to the predominance of positive or negative symptoms) during a working memory task. The results point to a different pattern of frontoparietal activities both comparing health × clinical groups and positive × negative schizophrenic groups. These results could be relevant to the understanding of the nature of working memory deficits in schizophrenic patients. Martinez et al. discussed the overlap between executive functioning in ADHD and Post Traumatic Stress Disorder presenting data concerning the overlap between neural mechanisms subserving executive functioning in those disorders.

In a developmental perspective, Zebdi et al. discussed the interaction between environmental influences, mainly parent-child relationships, and the development of the executive functioning and internalizing symptoms. The authors point to the importance of a research agenda exploring the interaction between those above cited factors. Medeiros et al. described executive functioning deficits in both aggressors and victims of Bullying. While aggressors seem to have deficits in hot executive functions, victims seem to be more impaired in cool executive functions. These differences are important to both comprehensions and to the prevention of the phenomena.

Concluding this research topic, three articles present data, which are directed to clinical practices. Considering assessment issues, Sediyama et al. showed psychometric properties of the Brazilian Version of the UPPS Impulsive Behavior Scale, the tool used to evaluate components of impulsivity. Venza et al. and Hadwin and Richards presented evidence of successful intervention to improve executive functions and another cognitive process in psychiatric patients. The former article present results of a cognitive training program, focused in reasoning stimulation, in a sample of adult bipolar patients. The later, present the result of a computer working memory training program used in a sample of adolescents with the high level of anxiety.

As proposed initially, this research topic presented a heterogenic scope including both theoretical and applied issues concerning executive functioning in psychiatric disorders. Far from offering an exhaustive exploration of the topic, we hope to present here some specific contributions on the state of the art of this essential theme.

## Author contributions

All authors listed have made a substantial, direct and intellectual contribution to the work, and approved it for publication.

### Conflict of interest statement

The authors declare that the research was conducted in the absence of any commercial or financial relationships that could be construed as a potential conflict of interest.
